# Circulating Immune Landscape Profiling in Psoriasis Vulgaris and Psoriatic Arthritis by Mass Cytometry

**DOI:** 10.1155/2024/9927964

**Published:** 2024-04-01

**Authors:** Xudong Sang, Tian Gan, Gai Ge, Dan Li, Youming Mei, Chun Pan, Siyu Long, Bibo Xie, Xiaobing Yu, Zhiming Chen, Hongsheng Wang

**Affiliations:** ^1^Zhejiang Institute of Dermatology, Deqing, China; ^2^Institute of Dermatology, Chinese Academy of Medical Sciences and Peking Union Medical College, Nanjing, China

## Abstract

**Background:**

Psoriasis, a systemic disorder mediated by the immune system, can appear on the skin, joints, or both. Individuals with cutaneous psoriasis (PsC) have an elevated risk of developing psoriatic arthritis (PsA) during their lifetime. Despite this known association, the cellular and molecular mechanisms underlying this progression remain unclear.

**Methods:**

We performed high-dimensional, in-depth immunophenotyping of peripheral blood mononuclear cells (PBMCs) in patients with PsA and psoriasis vulgaris (PsV) by mass cytometry. Blood samples were collected before and after therapy for a longitudinal study. Then three sets of comparisons were made here: active PsA *vs*. active PsV, untreated PsV *vs*. treated PsV, and untreated PsA *vs*. treated PsA.

**Results:**

Marked differences were observed in multiple lymphocyte subsets of PsA related to PsV, with expansion of CD4^+^ T cells, CD16^−^ NK cells, and B cells. Notably, two critical markers, CD28 and CD127, specifically differentiated PsA from PsV. The expression levels of CD28 and CD127 on both Naïve T cells (T_N_) and central memory CD4^+^ T cells (T_CM_) were considerably higher in PsA than PsV. Meanwhile, after treatment, patients with PsV had higher levels of CD28^hi^ CD127^hi^ CD4^+^ T_CM_ cells, CD28^hi^ CD127^hi^ CD4^+^ T_N_ cells, and CD16^−^ NK cells.

**Conclusion:**

In the circulation of PsA patients, the T_N_ and CD4^+^ T_CM_ are characterized with more abundant CD28 and CD127, which effectively distinguished PsA from PsV. This may indicate that individuals undergoing PsV could be stratified at high risk of developing PsA based on the circulating levels of CD28 and CD127 on specific cell subsets.

## 1. Introduction

Psoriasis, a systemic immune-mediated condition affecting the skin, joints, or both, is a global public health concern impacting approximately 3% of the world's population [1]. As the most prevalent form of cutaneous psoriasis (PsC), psoriasis vulgaris (PsV) is characterized by erythematous plaques with silvery scales attached [2]. Psoriatic arthritis (PsA) is now recognized as unique arthritis associated with PsC [3]. 75%–80% of PsA patients had skin lesions before arthritic involvement [4, 5]. A considerable portion of PsC patients may eventually develop PsA over time [6], often with a lag of 7–12 years between the onset of skin symptoms and arthritic involvement [7].

Currently, PsA lacks specific biomarkers, leading to a reliance on clinical evaluations and imaging findings for diagnosis. Regrettably, the therapeutic accomplishments seen in PsV have not been replicated in PsA. In phase III trials targeting TNF, the IL-23/IL-17 axis, or the JAK-STAT pathway, approximately half of PsA patients failed to meet the primary efficacy endpoint [8–10]. Psoriatic skin manifestations typically appear before joint symptoms by a period of 7–12 years [5], creating an opportunity for early intervention in high-risk groups of progression. Therefore, it is imperative to identify high-risk groups for preventative treatments.

On the topic of “PsV to PsA march”, many researchers have reported that clinical-demographic, genetic, and environmental risk factors (such as nail dystrophy, disease severity, and familial aggregation) contribute to the transition [11, 12]. Nevertheless, the exact biological mechanisms underlying this transition remain poorly understood. Given the differences in the immune landscape between PsV and PsA, it is likely that distinct immune profiles can be distinguished, offering insight into the immunopathology of disease progression.

In this context, we analyzed peripheral blood mononuclear cells (PBMCs) to characterize distinct immune signatures and discover new cell subsets through the high dimensionality of mass cytometry. We aim to find the disease-specific predictors of recognized PsA in PsV patients.

## 2. Methods

### 2.1. Patients and Samples

Peripheral blood specimens were collected from 19 psoriasis patients, including 12 PsV patients and seven PsA patients. Before sample collection, none of the study participants had received any treatment within the past 3 years. PsV diagnosis was based on characteristic clinical features and histological confirmation, while all PsA patients met the Classification Criteria for Psoriatic Arthritis (CASPAR) [13]. Seven PsV patients were matched with seven PsA patients based on their PASI (Psoriasis Area and Severity Index) score.  Table 1 summarizes the demographic and clinical characteristics of matched PsV and PsA patients, and *Supplementary 1* lists demographics for all study subjects. All patients received the same treatment regimen with adalimumab and underwent extensive clinical monitoring. All patients responded well to the treatment and no one experienced an adverse drug reaction. Blood samples were collected again after the symptoms have subsided and remained steady for 1 month. The study was carried out according to the Declaration of Helsinki, and informed consent was obtained from all study participants.

### 2.2. PBMCs Isolation

Fresh blood samples of patients were collected in K_2_EDTA-coated vacutainer tubes (BD Biosciences) and stored at 4°C until processed. PBMCs were separated using Ficoll–Paque PLUS (GE Healthcare) as operating instructions described. PBMCs were washed and resuspended in cell staining buffer (PBS with 0.5% BSA and 0.02% NaN_3_, CSB) to generate a single-cell suspension. Subsequently, cell counts were conducted using a hemocytometer in preparation for further staining.

### 2.3. Antibody Labeling

Antibodies were conjugated with the indicated metal tag using the MaxPAR Antibody Labeling kit (Fluidigm). The concentration of each antibody was examined using a Nano-100 spectrophotometer (ALLSHENG) after metal conjugation. After being diluted to 200 *μ*g/ml in Candor Antibody Stabilizer (Sigma–Aldrich), the conjugated antibodies were titrated for the optimal concentration for use.

### 2.4. Antibody Staining

For each sample, 1–3 × 10^6^ cells were washed with protein-free PBS and stained with 1 *μ*M Cisplatin for 5 min at room temperature. This was followed by staining the cell surface markers in CBS for 30 min at 4°C. Post staining, the cells were washed and kept at 4°C until acquisition.

### 2.5. CyTOF Data Acquisition

Cells underwent two rounds of washing with deionized water before the addition of EQ normalization beads containing Ce140, Eu151, Eu153, Ho165, and Lu175 (Fluidigm). The samples were then analyzed on a Helios instrument. After normalizing and randomizing values near zero through the Helios software, FCS files were uploaded to PLT Biological Information Analysis Team for analysis.

### 2.6. CyTOF Data Analysis

Debarcoding mass cytometry data involved employing a doublet filtration method utilizing mass-labeled barcodes, followed by manual gating to preserve viable, individual, authentic immune cells. To ensure precise immune subset data, either the FLOWSOM or X-shift algorithm was applied to all specimens. This approach took into account every cell event within each sample.

### 2.7. Statistical Analyses

According to normality distribution, group-to-group analyses were performed using the Mann–Whitney *U* test or two-sided unpaired *t*-test. Group differences in paired samples were assessed using the Wilcoxon matched-pairs signed-rank test. Categorical variables were compared using chi-square tests. Statistical analysis and visual representation of the data were conducted with the SPSS version24 and GraphPad Prism version 8.0. Significant differences were defined as *p* < 0.05.

## 3. Results

### 3.1. Comparisons of Circulating Immune Landscape between PsA and PsV

A cohort was set up with seven patients with active PsA and seven PASI-matched patients with active PsV. The baseline characteristics of the patients in the two groups were well-matched overall (Table 1). Peripheral blood samples from two groups of patients were processed immediately for mass cytometry. A phenotypic panel with 42 markers was utilized to differentiate and identify the primary immune cell populations. Then we clustered CD45^+^, ProMBP-1^−^, and CD66b^−^ cells to analyze immune cells excluding granulocytes.

**Figure 1:**
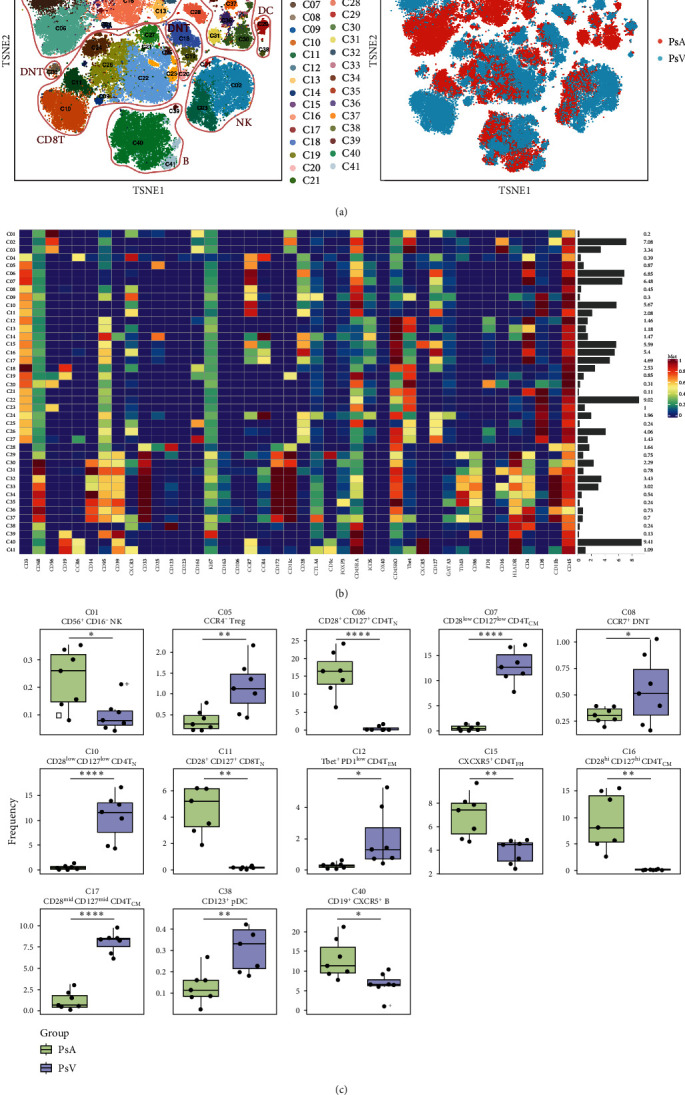
CyTOF analysis of circulating immune cell subsets in the active PsA group and active PsV group: (a) t-SNE plots showed 41 cell clusters of circulating immune cells (left) and distinct immune landscape of two groups of patients (right), (b) heatmap of the median arcsine transformed marker intensity normalized to a 0–1 range of the 42 phenotyping panel markers across the 41 annotated clusters, and (c) clusters with significant differences between the PsA group and PsV group. Data are shown as box plots extending from the 25^th^ to 75^th^ percentiles and the whiskers from the minimum to the maximum point; the middle line represents the median. *p* values were calculated using two-sided unpaired *t*-test or Mann–Whitney *U* test according to data distribution ( ^*∗*^ = *p* < 0.05,  ^*∗∗*^ = *p* < 0.01,  ^*∗∗∗*^ = *p* < 0.001,  ^*∗∗∗∗*^ = *p* < 0.0001). PsA, psoriatic arthritis; PsV, psoriasis vulgaris; NK, natural killer cell; Treg, regulatory T cell; T_N_, naïve T cell; T_CM_, central memory T cell; T_EM_, effector memory T cell; T_FH_, follicular helper T cell; and pDC, plasmacytoid dendritic cell.

We next carried out in-depth immunophenotyping by FlowSOM. By t-stochastic neighbor embedding (t-SNE), 41 cell clusters are merged and visualized in Figure 1(a). The heat map shown in Figure 1(b). further illustrates the expression levels of phenotypic markers in different populations. Those specific clusters with significant differences between PsA and PsV are shown in Figure 1(c).

### 3.2. Comparable Subsets of Lymphoid and Myeloid Cells in PsA and PsV

**Figure 2:**
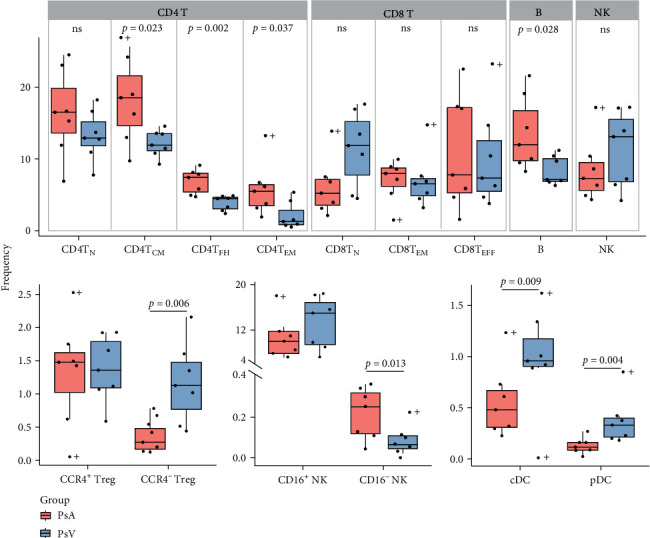
Comparisons of major immune cell subsets in the active PsA group and active PsV group. All *p* values were calculated using two-sided unpaired *t*-test or Mann–Whitney *U* test according to data distribution. TBNK, T, B, and NK-cell; T_N_, naïve T cell; T_CM_, central memory T cell; T_FH_, follicular helper T cell; T_EM_, effector memory T cell; T_EFF_, effector T cell; Treg, regulatory T cell; cDC, classical dendritic cell; pDC, plasmacytoid dendritic cell; and ns, no significance.

We observed clear differences in multiple lymphocyte subsets present in PsA compared with PsV (Figure 2). The expansion of CD4^+^ T cells (*p*=0.0323), especially central memory CD4^+^ T cells (T_CM_; *p*=0.0229) and circulating follicular helper T cells (cT_FH_; *p*=0.0019), were identified in PsA compared to PsV. However, CD4^+^ CCR4^−^ regulatory T cells (Treg; *p*=0.0060) were significantly decreased in patients with PsA. In addition, B cells (*p*=0.0379) and CD16^−^ NK cells (*p*=0.0129) were also significantly enhanced in PsA. No statistically significant frequency shift could be observed in CD8^+^ T cells.

By contrast, the two groups had less significant differences in myeloid cell subsets. Both classical dendritic cells (cDC; *p*=0.0096) and plasmacytoid dendritic cells (pDC; *p*=0.0041) had a significant decline in the PsA group. Monocytes, neither classical monocytes nor intermediate monocytes, did not change significantly.

### 3.3. Marked Differences in Expression Levels of Immune Markers between PsA and PsV

**Figure 3:**
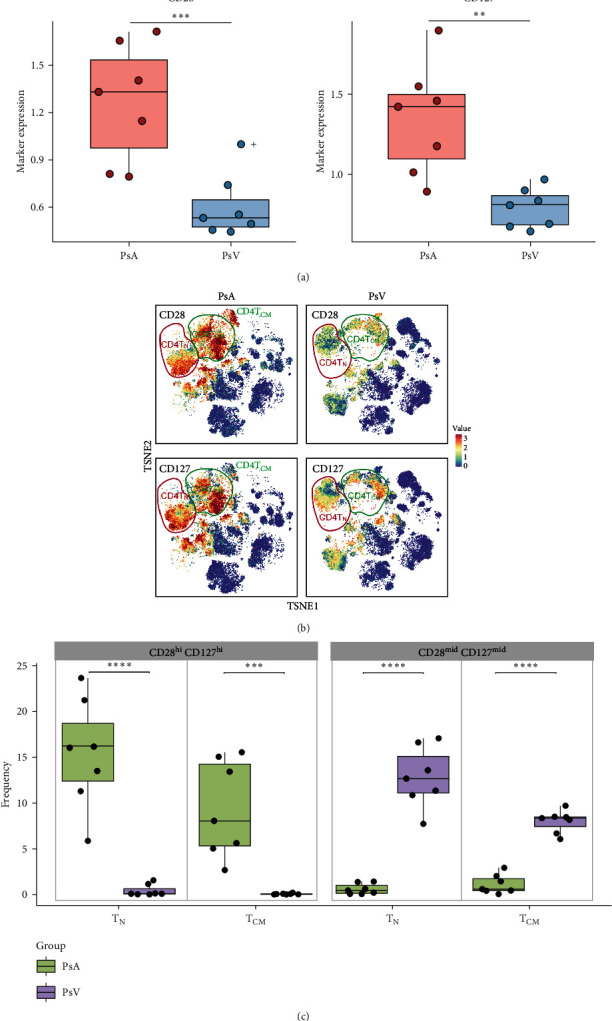
Comparisons of CD28 and CD127 between PsA and PsV: (a) comparisons of CD28 and CD127 marker expression levels in total circulating immune cells between PsA and PsV. *p* values were calculated using two-sided unpaired *t*-test ( ^*∗∗*^ = *p* < 0.01,  ^*∗∗∗*^ = *p* < 0.001), (b) t-NSE plots showed the identification of CD28 and CD127 in two groups. Red means high expression and blue means low expression, and (c) four subsets of CD4^+^ T_N_ and CD4^+^ T_CM_, divided by CD28 and CD127 high- or mid-expressing, were compared in PsA and PsV. *p* values were calculated using two-sided unpaired *t*-test ( ^*∗∗∗*^ = *p* < 0.001,  ^*∗∗∗∗*^ = *p* < 0.0001).

Next, the focus was turned toward the distinct signatures of immune marker expression. Of note, CD28 and CD127 were more abundant in PsA than in PsV (Figure 3(a)). Then we asked if any specific subpopulations were involved. Through t-SNE analysis, we found the Naïve CD4^+^ T cells (T_N_) and CD4^+^ T_CM_ were both characterized by a high level of CD28 and CD127 in the active PsA group, whereas, in PsV patients, the two markers were present at moderate or low levels (Figure 3(b)). Then, we used CD28 and CD127 to divide CD4^+^ T_N_ and CD4^+^ T_CM_ into high- and mid-expressing subsets, respectively, and compared these four cell subsets between PsA and PsV. Frequencies of all these subsets differed significantly between PsA and PsV (Figure 3(c)).

Moreover, in the PsA group, all of the chemokine receptor markers we identified either significantly increased or showed a tendency to increase. While CCR4 and CXCR3 exhibited a growing trend, both CCR6 and CXCR5 showed a statistically significant rise. In contrast, several cosignaling molecules, including TIM3, ICOS, and CD86, were lower in PsA (*Supplementary 2*).

### 3.4. Comparison of Circulating Immune Landscape from PsV Patients before and after Treatment

**Figure 4:**
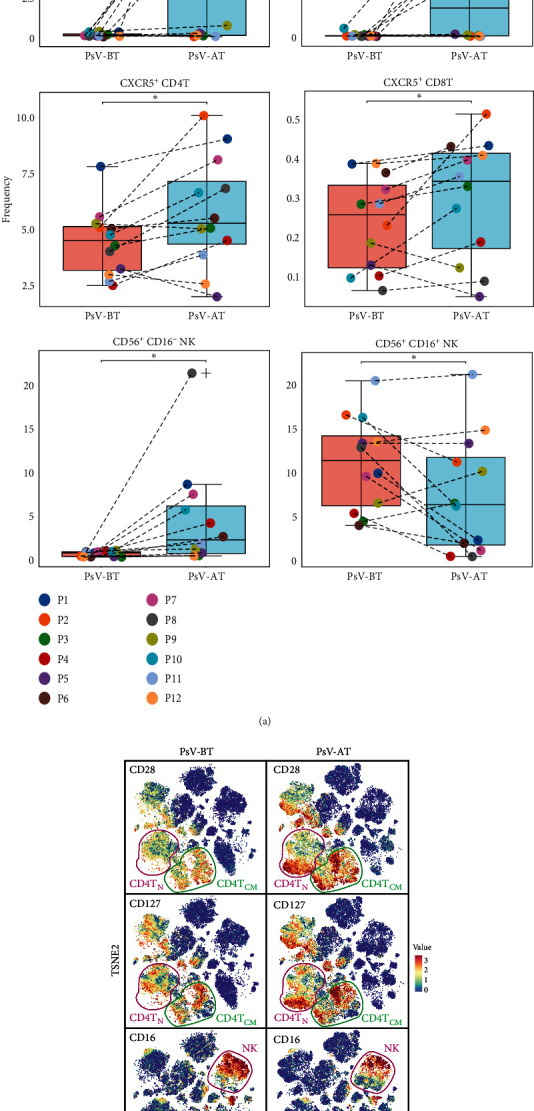
Cell subpopulations with significant differences between the PsV-BT group and PsV-AT group: (a) each individual was represented by two points corresponding to the before and after treatment state, with arrows connecting paired samples. *p* values were calculated using Wilcoxon matched-pairs signed rank test ( ^*∗*^ = *p* < 0.05,  ^*∗∗*^ = *p* < 0.01) and (b) t-NSE plots showed the identification of three markers (CD28, CD127, and CD16) in two groups. Red means high expression, and blue means low expression.

A new set of pairwise comparisons were performed here: untreated PsV *vs*. treated PsV. Blood samples from 12 patients with PsV before and after treatment were performed. By t-SNE, 34 cell clusters were merged and visualized in *Supplementary 3*. The expression levels of phenotypic markers for the various populations are further illustrated in a heat map shown in Figure 2(b). The comparison in frequencies of these 34 clusters is shown in *Supplementary 3*. Specific cell subsets with marked changes are shown in Figure 4.

After treatment, CD28^hi^ CD127^hi^ CD4^+^ T_CM_ cells (*p*=0.0122) and CD28^hi^ CD127^hi^ CD4^+^ T_N_ cells were significantly increased. In addition, CXCR5^+^ CD4 T cells and CXCR5^+^ CD8 T cells were both increased in the PsV-AT group. There was no significant change in the frequency of total NK cells, but CD16^−^ NK cells were increased while CD16^+^ NK cells declined in the PsA-AT group.

We found that the expression levels of CCR6, CCR7, FoxP3, CD28, and CD127 were significantly increased after treatment, while CD16 and CD45RO declined (*Supplementary 3*).

### 3.5. Comparison of Circulating Immune Landscape from PsA Patients before and after Treatment

Only three pairs of PsA blood samples were included here because of our loss to follow-up and the exclusion of the sample with abnormal duplicate results. By t-SNE, a total of 34 cell clusters were merged and visualized in *Supplementary 4*. The expression levels of phenotypic markers for the various populations are further illustrated in a heat map shown in Figure 3(b). The comparison in frequencies of these 31 clusters is shown in *Supplementary 4*. Due to the small sample size, no statistically significant differences were found among the 31 cell subsets we clustered. Thus, we interpret this result cautiously.

We noted two similar classical monocyte (cMO) subpopulations with slight differences in expression levels of CTLA-4 and TIM-3 differed clearly in frequencies before and after treatment. The CTLA-4 and TIM-3 levels on cMO were higher in the treated PsA group compared with the untreated group.

## 4. Discussion

In this study, a CyTOF analysis was performed on PBMCs from patients with PsA and PsV. Peripheral blood samples were collected before and after treatment for the longitudinal study. Three sets of pairwise comparisons were made: active PsV vs. active PsA, untreated PsV vs. treated PsV, and untreated PsA vs. treated PsA.

Elevated expression levels of CD28 and CD127 were observed in active PsA patients compared to active PsV patients in circulating CD4^+^ T_N_ and CD4^+^ T_CM_ subpopulations. The characteristics of PsA and PsV peripheral immune cells were clearly distinguished by the expression levels of CD28 and CD127 on CD4^+^ T_N_ and CD4^+^ T_CM_. CD28, known as a costimulatory molecule, is essential for the activation and expansion of T_N_ and T_CM_ cells [14]. Previous studies have shown that the proportion of CD4^+^ CD28^null^ T cells is inversely correlated with the severity of psoriasis. Furthermore, following medical improvement in nine individuals, the numbers of ex vivo CD4^+^ CD28^null^ T cells containing cytotoxic granules declined [15]. These findings have led to the development of abatacept, a fusion protein that antagonizes the CD28 costimulatory signal, inducing anergy in activated T-cells [16]. However, abatacept has shown effectiveness in treating PsA but limited efficacy in PsC therapy [16, 17], consistent with the findings of this study.

CD127, known as the IL-7 receptor *α* chain (IL-7R*α*), is activated by its ligand IL-7 to support the growth and sustenance of T cells, enhancing T-cell mediated immunity [18]. There is growing evidence that raised expression levels of IL-7 and CD127 are linked to various immune-mediated diseases such as rheumatoid arthritis and psoriasis. Colucci et al. [19] discovered elevated amounts of IL-7 in the blood and joint fluid of individuals with PsA. Using an anti-IL-7 functional antibody, they noted the dose-dependent reduction in osteoclast formation, indicating the crucial role of IL-7 in the osteoclast genesis in PsA [19]. In addition, the other ligand for CD127, thymic stromal lymphopoietin (TSLP), has been found statistically increase in psoriasis patients and correlated with severity of psoriasis [20, 21]. El-Ghareeb et al. [21] reported that serum TSLP levels were significantly elevated in PsA compared to PsC, supporting our observation that CD127 was more abundant in PsA than in PsV.

We also observed an unexpected phenomenon in the treated PsV group. The expression levels of CD28 and CD127 were much higher in treated PsV patients compared to those who did not receive treatment. Specifically, two subsets of CD4^+^ T_CM_ and CD4^+^ T_N_, expressing high levels of CD28 and CD127, were considerably enriched in the treated PsV group, whereas it was barely detectable before treatment. This upregulation may be a result of a compensatory mechanism triggered by immunosuppressive agents. It may point to the risk of arthritis in patients with PsV, even if their skin symptoms show improved.

A significant increase in CXCR5^+^ CD4^+^ memory T cells (CXCR5^+^ CCR7^+^ PD1^–^ ICOS^−^ CD45RO^+^ CD28^+^ CD127^+^ CD4^+^) was observed in active PsA patients compared to PsV patients, as well as in the PsV-AT group compared to PsV-BT group. These cells, containing long-lived memory cells and sharing functional properties with T_FH_ [22], are currently referred to as circulating memory T_FH_ (cT_FH_) [23]. Furthermore, cT_FH_ cells can be categorized into active subset (ICOS^+^ PD-1^++^ CCR7^low^) and quiescent subsets (ICOS^−^ PD-1^+^ CCR7^int^ and ICOS^−^ PD-1^−^ CCR7^hi^). The subset we observed was quiescent memory cT_FH_. The phenotype of cT_FH_ cells has been extensively studied in various autoimmune diseases, such as rheumatoid arthritis [24]. Niu et al. [25] reported a significant increase in the frequency of cT_FH_ cells in psoriasis, which was positively correlated with disease severity. However, there is a limited number of published studies investigating the relationship between PsA and cTFH cells, suggesting a potential area for future research.

The proportions of CD4^+^ T major subpopulations were increased in the PsA group compared to the PsV group, while CCR4^−^ CD4^+^ T_regs_ decreased significantly. CCR4^−^ Tregs, an atypical subset of T_regs_, are believed to be linked to highly activated, short-lived, terminally differentiated T_regs_. Functionally, CCR4^−^ T_regs_ are less immunosuppressive than classical CCR4^+^ T_regs_ [26]. Recent studies suggest that T_regs_ are dysfunctional in most psoriasis patients [27]. Although the significance of T_reg_ frequencies is not fully understood, it is established that attenuated T_reg_ function and disrupted Th17/T_reg_ balance play a role in the pathogenesis and worsening of psoriasis. Our result suggests that the imbalance in T_reg_ frequency or functionality offers a new perspective to distinguish PsA from PsV.

An expansion of CD56^bright^ CD16^−^ NK cells was observed in PsA patients compared to PsV patients. Additionally, in the treated PsV group, CD16^−^ NK cells showed a significant increase, while CD16^+^ NK cells exhibited an overall decline. Previous studies have identified two distinct populations of human NK cells: CD56^dim^CD16^+^ NK cells and CD56^bright^ CD16^dim/−^ NK cells [28]. The CD56^dim^CD16^+^ NK cell subset is primarily known for its cytotoxic functions, whereas the CD56^bright^ CD16^dim/−^ NK cell subset is capable of producing a variety of cytokines comparable to CD4^+^ T helper cell subsets, including IFN-*γ*, TNF-*α*, IL-10, IL-13, and GM-CSF [29]. Some studies revealed altered levels of CD56^dim^ CD16^+^ and CD56^bright^ CD16^−^ NK cell subsets in the peripheral blood of psoriasis patients compared to healthy donors, suggesting a potential role of NK cells in psoriasis pathogenesis. However, conflicting results have been reported by other studies [30], indicating the need for further research to establish a definitive link between NK cells and psoriasis pathology.

Plasmacytoid dendritic cells (pDCs) represent a rare dendritic cells (DCs) subset characterized by a plasma cell-like morphology and a distinct surface phenotype (CD4^+^ CD45RA^+^ CD123^+^ HLA-DR^+^). Typically, in homeostatic conditions, pDCs are not found in undisturbed skin or peripheral tissues. An essential aspect of the innate immune response in psoriatic inflammation is the release of type I IFN by activated pDCs [31]. We have observed a notable decrease in circulating pDCs in PsA patients. This decrease aligns with the results of Jongbloed et al. [32], who also reported a reduction in circulating pDCs in PsA patients compared to healthy individuals. This reduction may indicate the migration of pDCs from the bloodstream to the inflamed synovial compartment.

In the PsV-AT group, there is a notable expansion of a new subgroup of CD8^+^ T cells (CXCR5^+^ PD-1^mid^ T-bet^+^ CD8^+^). Recent studies have identified distinctive populations of CD8^+^ T cells that express CXCR5, referred to as follicular cytotoxic T cells (Tfc), in various experimental systems and health conditions [33]. However, the exact function of Tfc is still not clearly defined. Previous studies have shown that these cells can act in various capacities depending on the conditions. Some studies suggest that Tfc cells may function as regulatory T cells that help prevent the development of autoantibodies [34], which aligns with our findings. Nevertheless, the specific role of this subset in PsV remains uncertain and requires further experimental validation

This study has several limitations. The analysis was carried out on a small, heterogeneous sample, highlighting the need for future research with a larger patient cohort to validate our results. Furthermore, the lack of a healthy control group hinders the assessment of whether treated patients have fully recovered or are still experiencing compensated inflammation. Last but not least, functional verification of those cell subsets and molecules is still required.

In summary, we have used CyTOF analysis to profile the circulating immune landscape in PsV and PsA. Our study revealed significant differences in circulating immune cell populations and molecules between PsA and PsV, as well as before and after treatment within each disease group. Notably, our findings suggest that monitoring CD28 and CD127 expression levels on CD4^+^ T_CM_ and CD4^+^ T_N_ could be crucial in identifying psoriasis patients at high risk of developing arthritis. Additionally, we identified several novel immune cell subsets with unknown functions, highlighting the need for further research to fully comprehend their roles.

## Figures and Tables

**Table 1:** Demographics and clinical features of the active PsA group and active PsV group.

Clinical feature	Active PsA (*n* = 7)	Active PsV (*n* = 7)	*p*-Value
Demographics			
Age, mean ± SD (years)	43.9 ± 11.7	36.7 ± 11.0	0.264^a^
Gender, male (%)	4 (57.14)	5 (71.43)	0.999^b^
Clinical assessments			
PASI			
Mean ± SD	7.94 ± 4.20	7.90 ± 4.16	0.985^a^
Mild (<3)	2	2	—
Moderate (3–10)	2	2	—
Severe (<10)	3	3	—
TJC (0–66)	2 (1–10)	/	/
SJC (0–66)	2 (1–7)	/	/
Pain VAS (0–10)	4 (2–5)	/	/

Abbreviations: PASI, psoriasis area and severity Index; TJC, tender joint count; SJC, swollen joint count; and VAS, visual analogue scale. ^a^: *t*-test. ^b^: Fisher's exact test.

## Data Availability

Data available within the article or its supplementary materials. Raw data fully available without restriction.

## Supplementary Materials

Supplementary 1 Table S1: information of patients in this study.

Supplementary 2 Figure S1: comparisons of markers expression levels between PsA and PsV.

Supplementary 3 Figure S2: CyTOF analysis of circulating immune cell subsets in the PsV-BT group and PsV-AT group.

Supplementary 4 Figure S3: CyTOF analysis of circulating immune cell subsets in the PsA-BT group and PsA-AT group.
