# Supplementary material for: Circulating Immune Landscape Profiling in Psoriasis Vulgaris and Psoriatic Arthritis by Mass Cytometry
Source: J Immunol Res. 2024 Apr 1;2024:9927964. doi: 10.1155/2024/9927964 (PMC11001477; doi:10.1155/2024/9927964)
Supplement: Supplementary 1 — Table S1: information of patients in this study. [file 9927964.f1.docx]

**Supplementary Table 1. Information of patients in this study**

| **Donor ID** | **Age** | **Gender** | **PASI** | | | **Duration of disease (Months)** |
| --- | --- | --- | --- | --- | --- | --- |
|  |  |  | **T0** | **T1** | **T2** |  |
| PSA01 | 48 | F | 10.6 | 9.25 | 7.2 | 7 |
| PSA02 | 54 | M | 2 | 1 | 0.46 | 2 |
| PSA03 | 39 | M | 8.25 | 5.6 | 4.2 | 5 |
| PSA04 | 57 | M | 2.9 | 2.2 | 1.3 | 3 |
| PSA05 | 50 | F | 11.7 | 10.4 | 3.18 | 3.5 |
| PSA06 | 35 | M | 7.35 | 4.2 | 1.75 | 2.5 |
| PSA07 | 47 | F | 12.8 | 9.15 | 3.75 | 4 |
| PSV01 | 24 | F | 2.55 | 1.3 | 0.26 | 2 |
| PSV02 | 34 | M | 6.55 | 0.8 | 0 | 1.5 |
| PSV03 | 32 | M | 2.75 | 0.93 | 0 | 1.5 |
| PSV04 | 45 | M | 9 | 8 | 4.5 | 5 |
| PSV05 | 36 | M | 12 | 5.3 | 1.7 | 2.5 |
| PSV06 | 29 | F | 10.7 | 5.3 | 2.2 | 3.5 |
| PSV07 | 33 | M | 12.65 | 9.45 | 5.65 | 5 |
| PSV08 | 50 | M | 7.8 | 5.6 | 2.7 | 4 |
| PSV09 | 32 | F | 26.05 | 8.2 | 4.2 | 4.5 |
| PSV10 | 39 | M | 12.55 | 5.75 | 3.1 | 3.5 |
| PSV11 | 30 | F | 3.75 | 2.3 | 0.83 | 2 |
| PSV12 | 21 | M | 26.6 | 20.75 | 1.55 | 3 |

**T0: onset of disease, before treatment; T1: 1 month after treatment; T2: 2 months after treatment**
