# Supplementary material for: Circulating Immune Landscape Profiling in Psoriasis Vulgaris and Psoriatic Arthritis by Mass Cytometry
Source: J Immunol Res. 2024 Apr 1;2024:9927964. doi: 10.1155/2024/9927964 (PMC11001477; doi:10.1155/2024/9927964)
Supplement: Supplementary 2 — Figure S1: comparisons of markers expression levels between PsA and PsV. [file 9927964.f2.pdf]

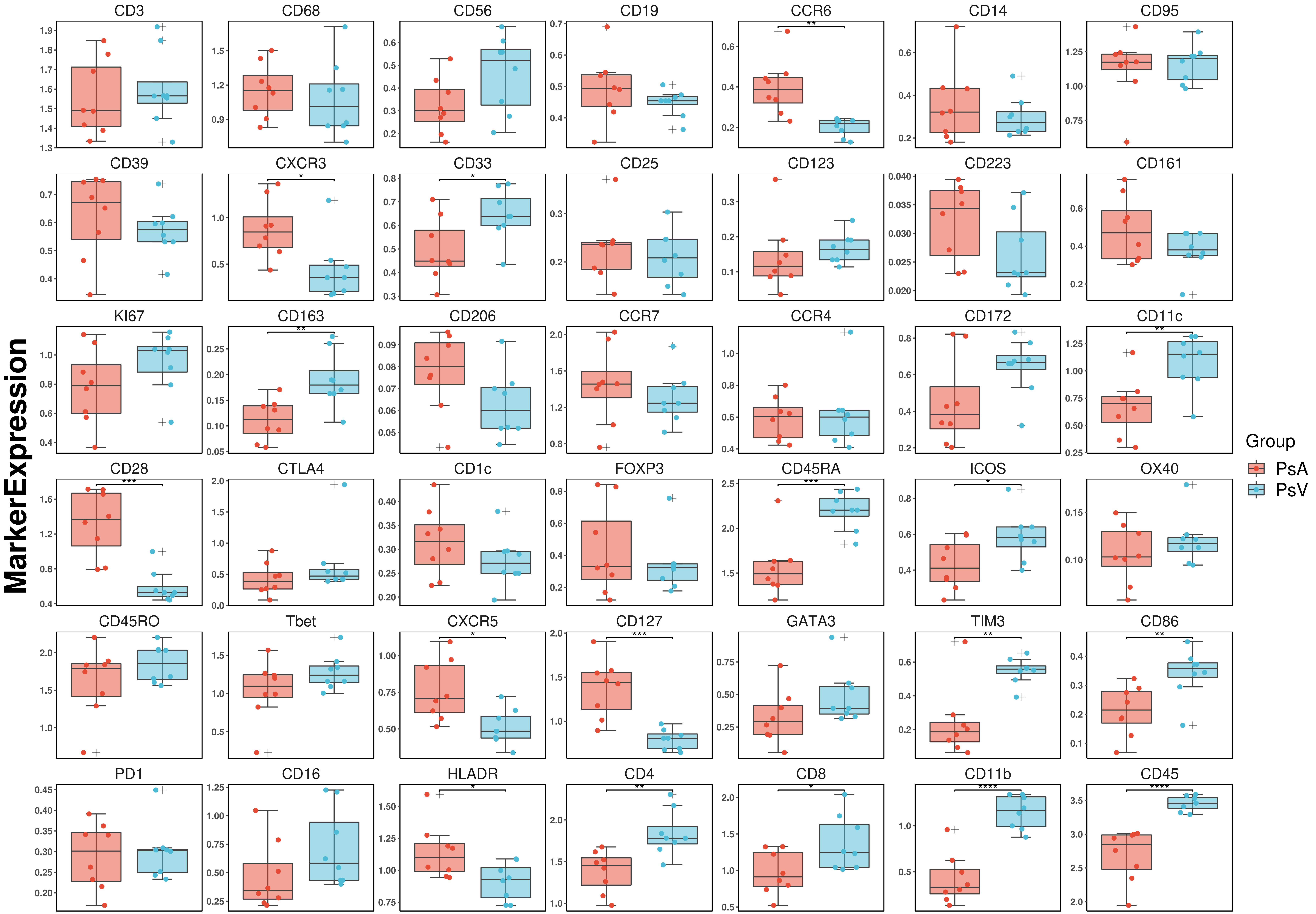

Supplementary Fig. 1 Comparisons of markers expression levels between PsA and PsV.
p values were calculated using two-sided unpaired t-test or Mann-Whitney U test according to data distribution (* = p < 0.05, ** = p < 0.01, *** = p < 0.001).
