# Supplementary material for: Circulating Immune Landscape Profiling in Psoriasis Vulgaris and Psoriatic Arthritis by Mass Cytometry
Source: J Immunol Res. 2024 Apr 1;2024:9927964. doi: 10.1155/2024/9927964 (PMC11001477; doi:10.1155/2024/9927964)
Supplement: Supplementary 3 — Figure S2: CyTOF analysis of circulating immune cell subsets in the PsV-BT group and PsV-AT group. [file 9927964.f3.docx]

**(a)**


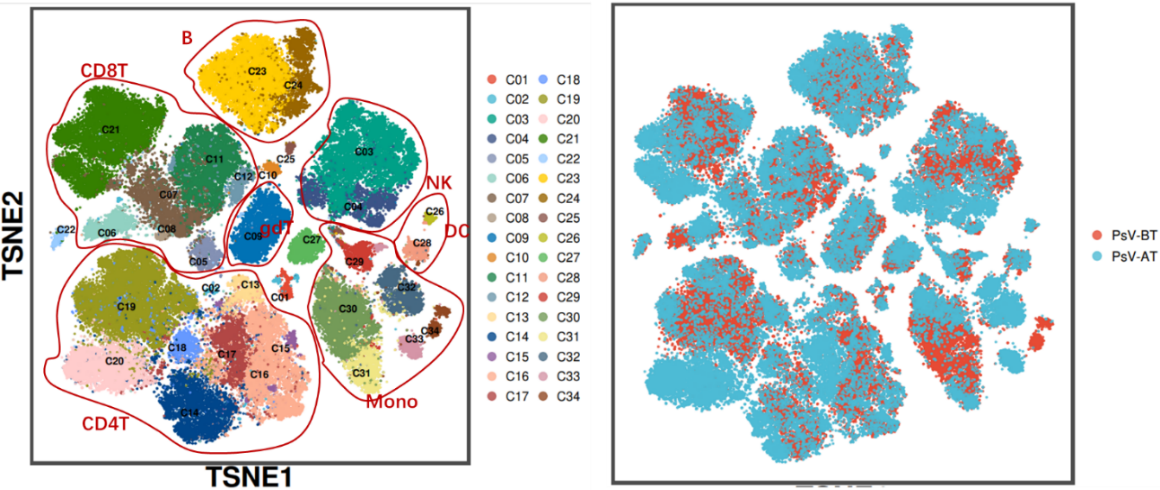


**(b)**

**
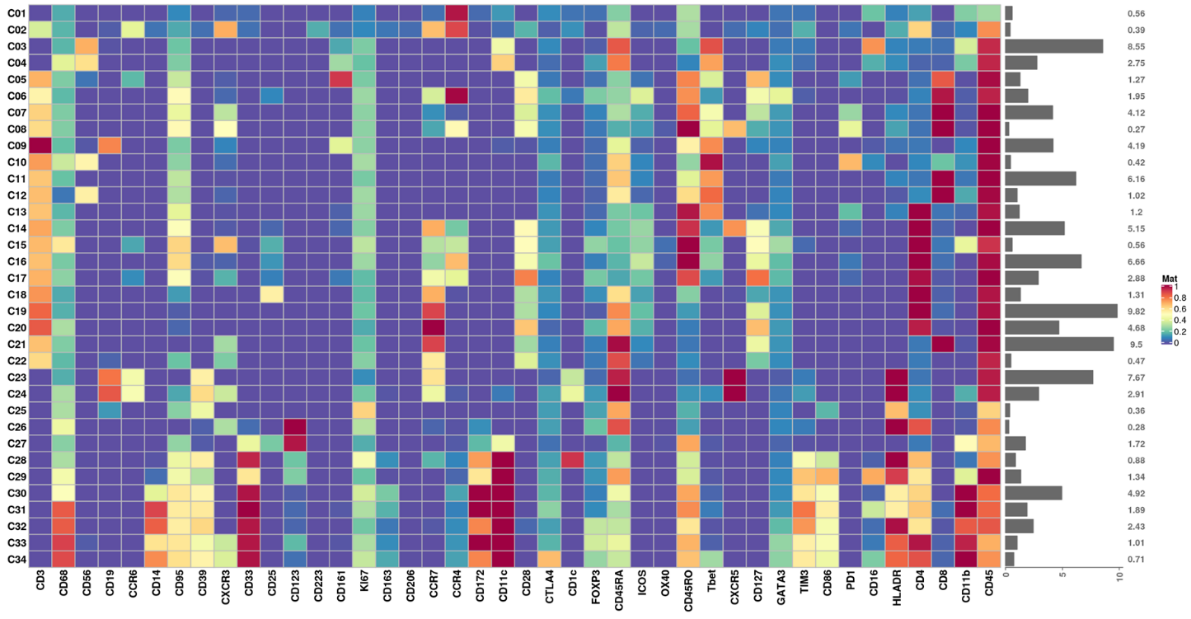
**

**(c)**

**
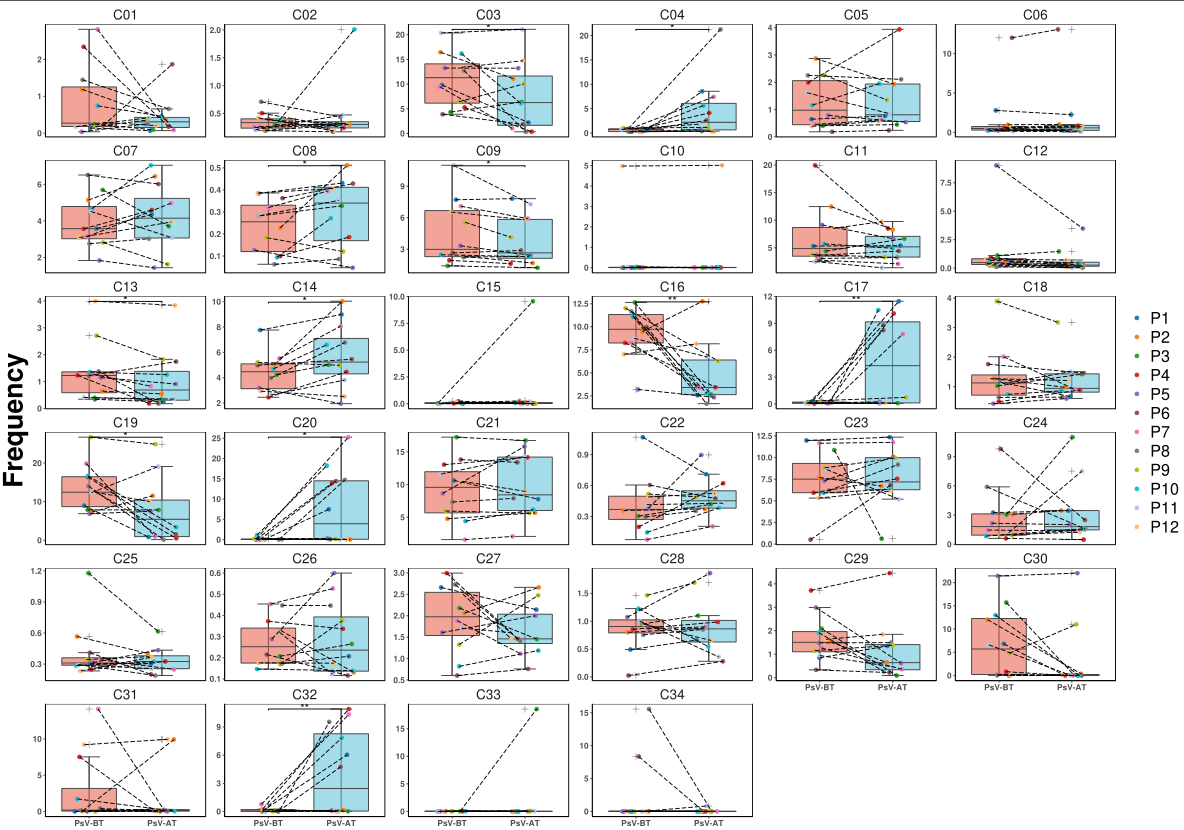
**

**(d)**

**
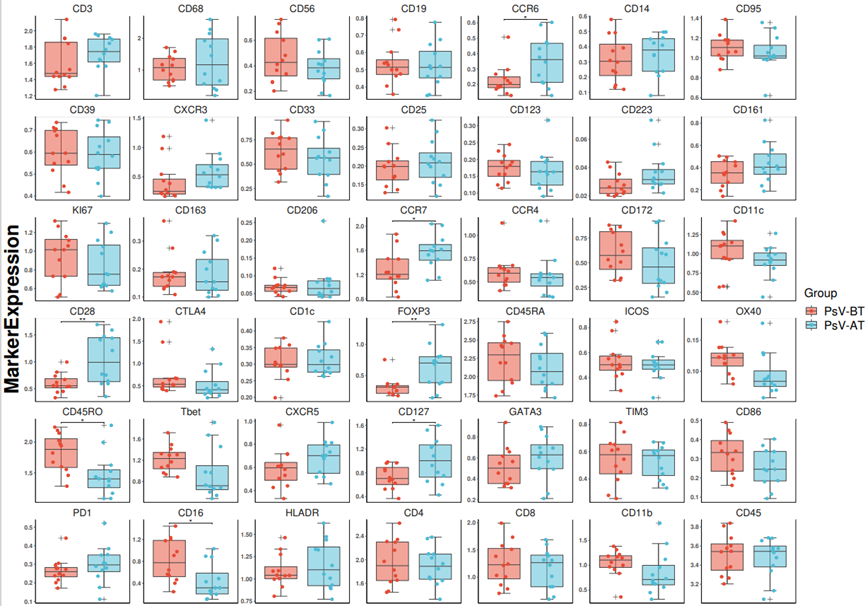
**

**Supplementary Fig. 2 CyTOF analysis of circulating immune cell subsets in the PsV-BT group and PsV-AT group.**

(a) t-SNE plots showed 34 cell clusters of circulating immune cells (left) and distinct immune landscape of two groups of patients (right).

(b) Heatmap of the median arcsine transformed marker intensity normalized to a 0–1 range of the 42 phenotyping panel markers across the 34 annotated clusters.

(c) Comparisons in frequencies of 41 clusters between the PsV-BT group and PsV-AT group.

(d) Comparisons of markers expression levels between the PsV-BT group and PsV-AT group.

All p values were calculated using two-sided paired t-test or Mann-Whitney U test according to data distribution (* = p < 0.05, ** = p < 0.01, *** = p < 0.001).

PsV-BT, psoriasis vulgaris patients before treatment; PsV-AT, psoriasis vulgaris patients after treatment.
