# Supplementary material for: Circulating Immune Landscape Profiling in Psoriasis Vulgaris and Psoriatic Arthritis by Mass Cytometry
Source: J Immunol Res. 2024 Apr 1;2024:9927964. doi: 10.1155/2024/9927964 (PMC11001477; doi:10.1155/2024/9927964)
Supplement: Supplementary 4 — Figure S3: CyTOF analysis of circulating immune cell subsets in the PsA-BT group and PsA-AT group. [file 9927964.f4.docx]

**(a)**

**
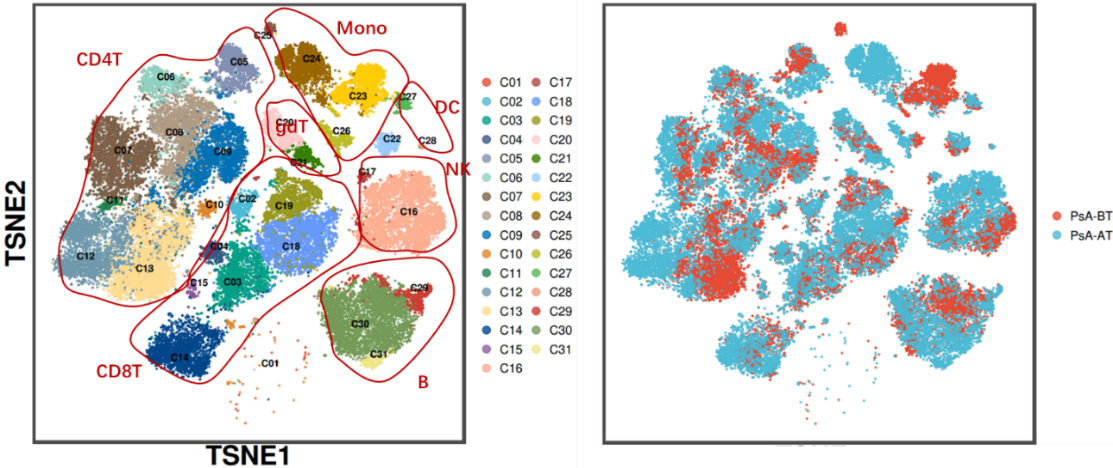
**

**(b)**

**
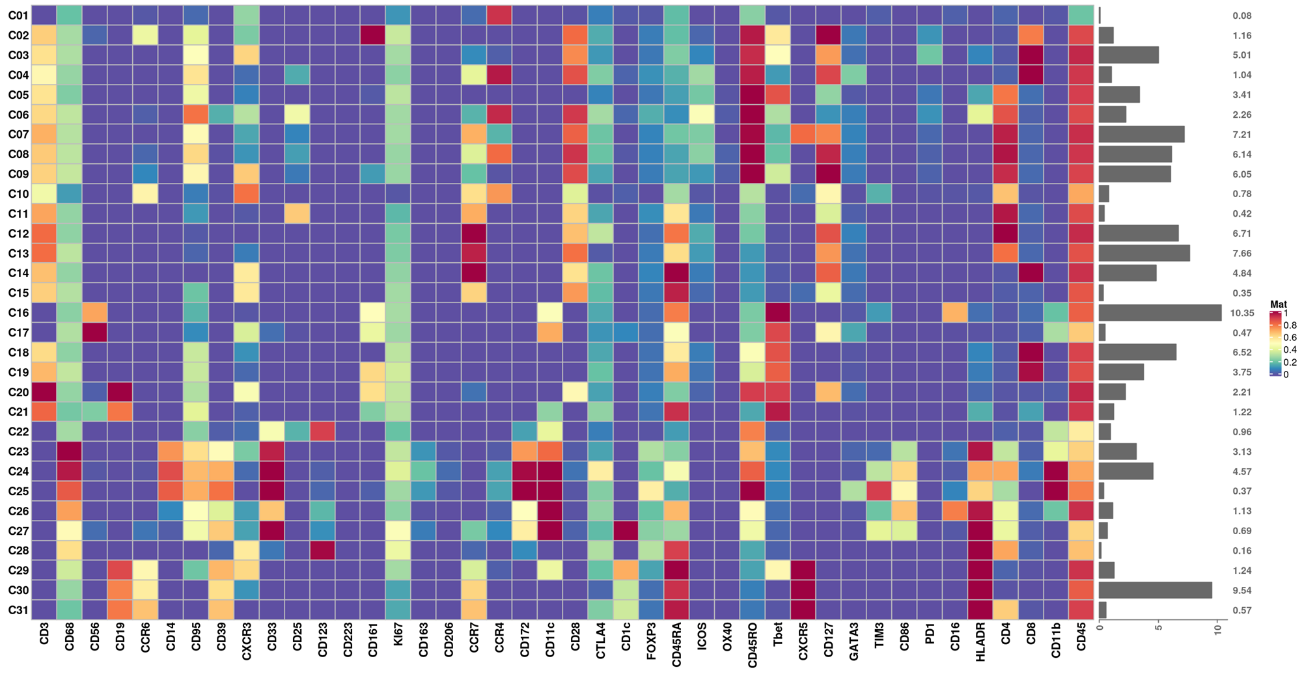
**

**(c)**

**
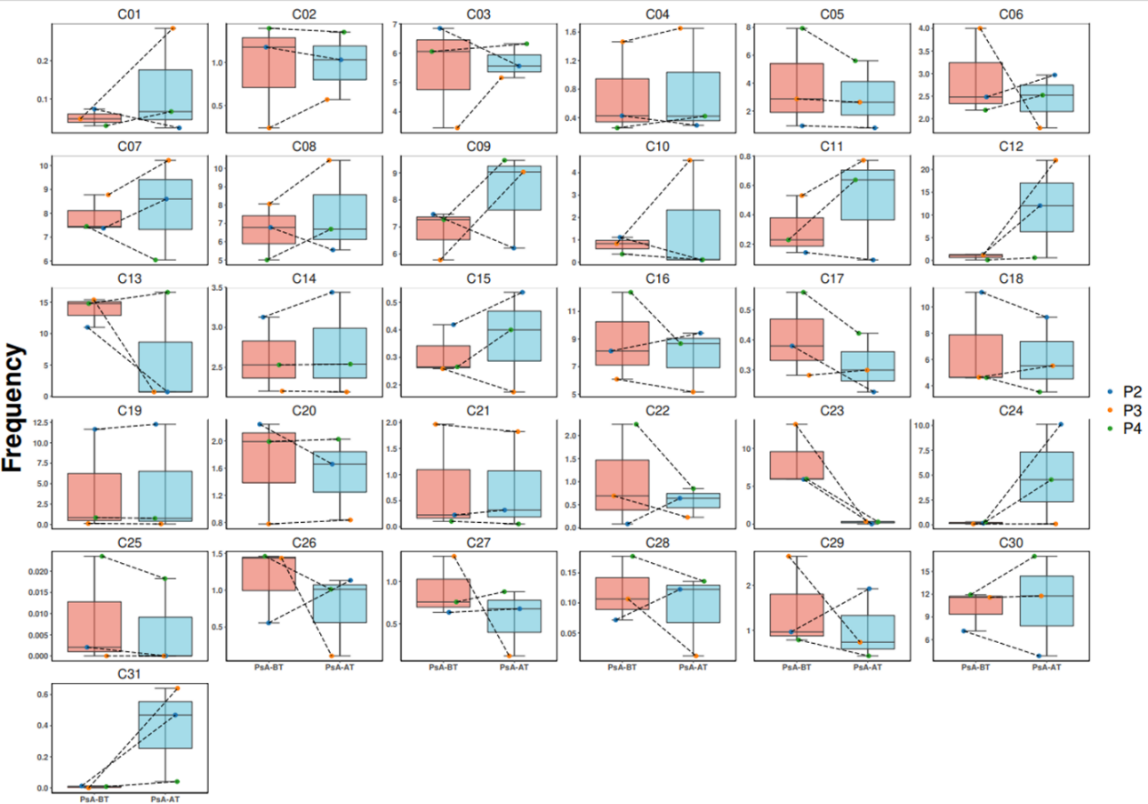
**

**Supplementary Fig 3. CyTOF analysis of circulating immune cell subsets in the PsA-BT group and PsA-AT group.**

(a) t-SNE plots showed 31 cell clusters of circulating immune cells (left) and distinct immune landscape of two groups of patients (right).

(b) Heatmap of the median arcsine transformed marker intensity normalized to a 0–1 range of the 42 phenotyping panel markers across the 31 annotated clusters.

(c) Comparison of 31 cell clusters frequencies between PsA-BT group and PsA-AT group. All p values were calculated using paired Wilcox test.

PsA-BT, psoriasis arthritis patients before treatment; PsA-AT, psoriasis arthritis patients after treatment.
